# Unilateral Paracondylar-Epitransverse Neo-Articulation with Secondary Atlas-Axis Rotation Anomaly: Teaching point: Variants of the craniovertebral junction can be depicted and characterized on CT

**DOI:** 10.5334/jbsr.1844

**Published:** 2019-06-28

**Authors:** Nick Janssen, Wouter Mebis, Jan Gielen

**Affiliations:** 1 University of Antwerp, BE; 2 Antwerp University Hospital, BE

**Keywords:** paracondylar process, epitransverse process, computed tomography, atlas, axis, rotation

## Case

**Figure 1:**
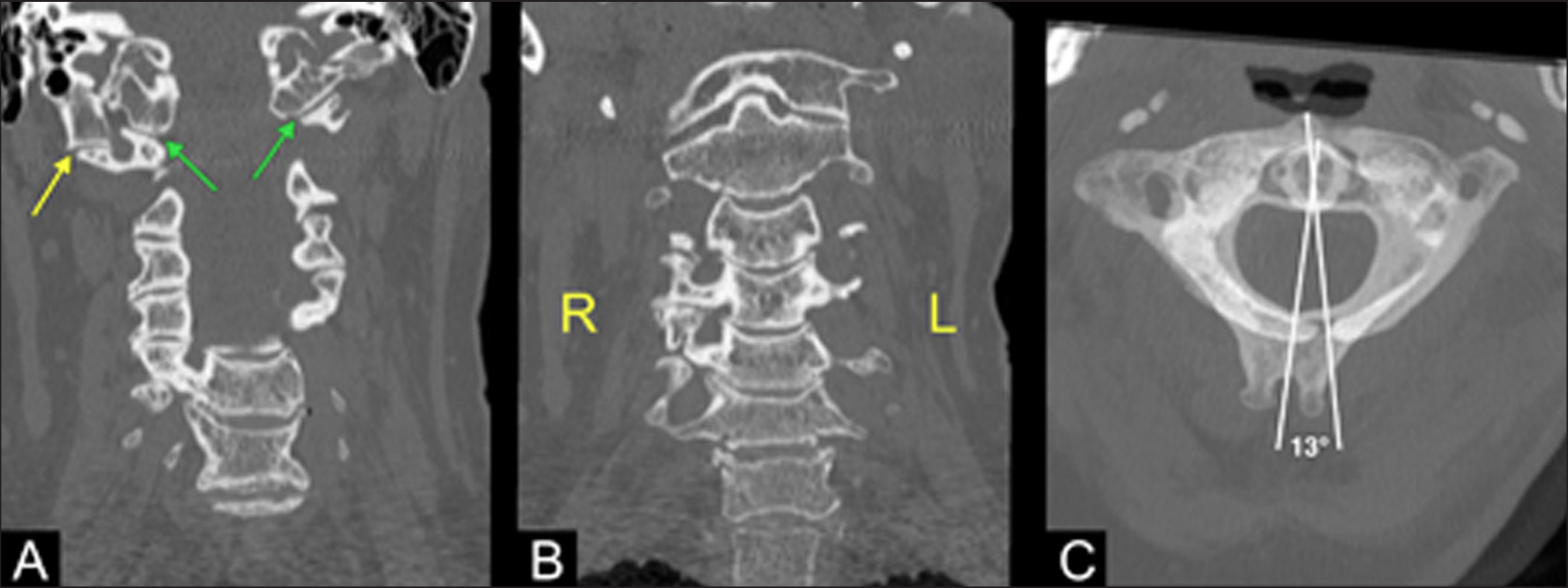


**Figure 2:**
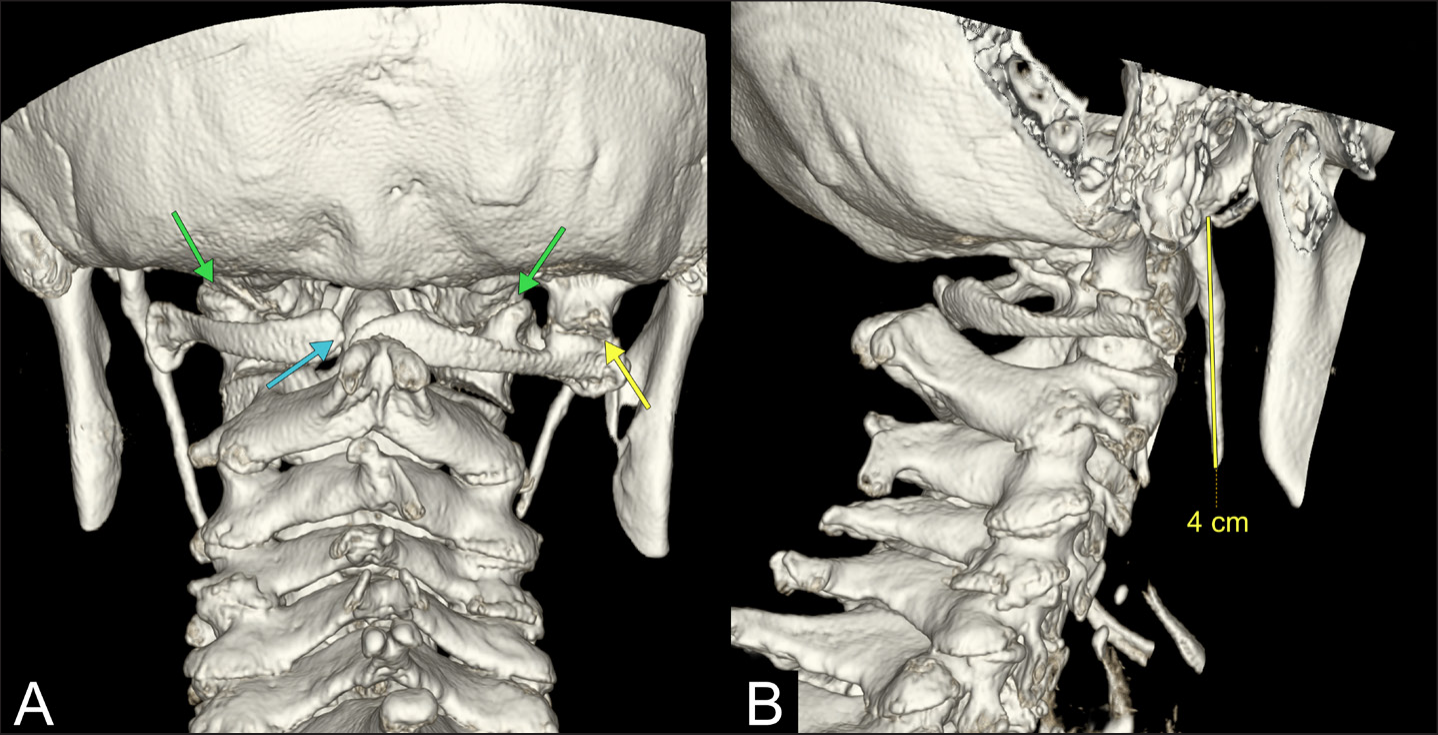


A 59-year-old woman presented eight months after whiplash trauma with persisting cervical pain. Radiography of the cervical spine showed no traumatic lesions (not shown). Computed tomography (CT) and 3D reconstruction of the cervical spine showed an incidental asymmetrical craniocervical junction with a supernumerary occipital condyle on the right (Figures 1A and 2A, yellow arrow), lateral to the proper occipital condyle (Figures 1A and 2A, green arrow). This extra condyle articulated with a hypertrophic right transverse process of the atlas. Signs of neo-articulation with degenerative changes such as subchondral sclerosis, osteophytosis, and dystrophic calcifications are documented. Other findings included an unfused posterior arch of C1 (Figure 2A, blue arrow), rotation of C2 on C1 (13°) despite the neutral position of the head (Figure 1C), a mild sinistroconvex cervical scoliosis (Cobb angle 11°), and an asymmetrical right-sided uncovertebral degeneration from C3 to Th2 (Figure 1B). Furthermore, an elongation of both styloid processes (approximately 4 cm) was also present (Figure 2B).

## Comment

The normal atlanto-occipital articulation consists of two condyloid synovial joints that connect the occiput with the first cervical vertebra and are reinforced by a fibrous capsule.

In this case we have a combination of two rare anatomical variants occurring together and forming an accessory joint on the right side:

1. The *paracondylar process*: a variant of the occipital bone where an enlarged bony process extends caudally from the paracondylar region towards the transverse process of the atlas. This can occur bilateral or unilateral, as in this case.
2. The *epitransverse process*: a variant of the atlas where a bony outgrowth arises from the transverse process and articulates with the occiput, or in this case with the paracondylar process.

These variants are generally of little clinical importance and usually incidental findings. Reported prevalence ranges from 0.077% to 0.29%. Large process size and fusion or articulation with the epitransverse process may cause occipitocervical pain, functional limitations of head and neck movement or even torticollis. Some clinical case reports suggest resection of the process (or joint) to relieve symptoms [1].

This anatomical variant is easily missed on the anteroposterior and lateral cervical radiographs due to bony superposition but is accurately depicted on CT-imaging of the cervical spine.

Unique to this case is the concurrent rotation between C2 and C1, which is thought to be a result of the accessory joint degeneration. Likewise, there might be a relation between the accessory joint and the mild scoliosis and asymmetrical degenerative changes in the cervical spine due to increased mechanical stress. Paracondylar and epitransverse processes can be found in conjunction with other anomalies of the occipitocervical junction and skull base which can be explained by the common embryologic origin. The specific co-existence of the paracondylar-epitransverse articulation, unfused posterior arch of C1 and elongated styloid processes has not been reported to date.
